# The role of SH3BP2 in the pathophysiology of cherubism

**DOI:** 10.1186/1750-1172-7-S1-S5

**Published:** 2012-05-24

**Authors:** Ernst J Reichenberger, Michael A Levine, Bjorn R Olsen, Maria E Papadaki, Steven A Lietman

**Affiliations:** 1 University of Connecticut Health Center, Department of Reconstructive Sciences, Center for Regenerative Medicine and Skeletal Development, Farmington, CT, USA; 2 Division of Endocrinology and Diabetes, The Children’s Hospital of Philadelphia and Department of Pediatrics, University of Pennsylvania School of Medicine, Philadelphia, PA, USA; 3 Department of Developmental Biology, Harvard School of Dental Medicine, Boston, MA, USA; 4 Department of Oral and Maxillofacial Surgery, Massachusetts General Hospital, Harvard School of Dental Medicine, Boston, MA, USA; 5 The Departments of Orthopaedic Surgery and Biomedical Engineering, Cleveland Clinic Lerner Research Institute, Cleveland, OH, USA

## Abstract

Cherubism is a rare bone dysplasia that is characterized by symmetrical bone resorption limited to the jaws. Bone lesions are filled with soft fibrous giant cell-rich tissue that can expand and cause severe facial deformity. The disorder typically begins in children at ages of 2-5 years and the bone resorption and facial swelling continues until puberty; in most cases the lesions regress spontaneously thereafter. Most patients with cherubism have germline mutations in the gene encoding SH3BP2, an adapter protein involved in adaptive and innate immune response signaling. A mouse model carrying a Pro416Arg mutation in SH3BP2 develops osteopenia and expansile lytic lesions in bone and some soft tissue organs. In this review we discuss the genetics of cherubism, the biological functions of SH3BP2 and the analysis of the mouse model. The data suggest that the underlying cause for cherubism is a systemic autoinflammatory response to physiologic challenges despite the localized appearance of bone resorption and fibrous expansion to the jaws in humans.

## Introduction

“Bone dystrophies paint queer and irregular pictures throughout the skeleton and have been reported in most bones” W.A. Jones begins his 1950 review, where he proposed the name “cherubism” for the multilocular cystic disease of the jaws that he had first described 17 years earlier [1,2]. In 2011 we still lack good explanations for the bilateral expression of cherubism [MIM 602104] lesions. Other areas of investigation are the limitation of the aggressive bone resorption and expansion of fibrous tissues in the maxilla and mandible as well as the age-dependent onset in children at age 2-5 years, and in most cases the spontaneous regression of the fibrous growths after puberty [3]. Cherubism typically begins with a swelling of submandibular lymph nodes. The phenotype comes to the attention of health care providers, often dentists, at its early stages when excessive bone resorption in the jaws causes characteristic symmetrical cystic lesions that can be detected by routine panoramic radiographs. The “cherubic” swelling of cheeks occurs when the fibrous tissue filling the cysts expands and deforms the cortical shell.

Clinical management of cherubism has progressed significantly but therapeutic approaches to inhibit or delay the progression of cherubic lesions are not available. The gaps in our understanding of the natural history of cherubism, and the molecular mechanism that initiates and maintains bone resorption as well as the replacement of bone with tumor-like fibrous tissue are now being addressed by several research groups. In this review we will assess the many functions of the cherubism gene *SH3BP2* [MIM 118400] in immune cells and osteoclasts and discuss how animal models and in vitro studies can help to understand the human disease.

## *SH3BP2*: genetic aspects

Cherubism is classically transmitted as an autosomal dominant trait, but there are indications that a recessive form may also exist. Based on a thorough statistical analysis of 21 previously published families by Anderson and McClendon, 100% penetrance in males and reduced penetrance (70 - 50%) in females has been reported [4]. However, the authors concede in this retrospective study that only 50% of the adult female family members which were considered unaffected underwent radiographic examination. The apparently reduced female penetrance may also be due to examination of some children before they developed clinical signs of cherubism. Unfortunately, this paper has been cited many times since then without acknowledging these caveats. In the experience of our group, we cannot confirm incomplete penetrance but we have seen variable expressivity within families. It should be noted that older patients with a mild form of cherubism may have bone lesions that have been remodeled with normal mandibular bone and therefore signs of cherubism may no longer be detected by radiographs [5]. Based on published case reports of cherubism as well as patients referred to our clinics and research environment there appears to be no obvious difference in the prevalence of the disorder among different racial or ethnic groups. Adequate epidemiologic data for cherubism do not exist.

Approximately 50% of cases seen in our laboratory at UCHC are sporadic and represent de novo mutations. The genetic interval for the autosomal dominant form of cherubism was first identified in 1999 by linkage and haplotype analysis to be on chromosome 4p16.3 [6,7]. The 1.5 Mb cherubism locus is contained within the locus for Wolf-Hirschhorn disease [8].

Wolf-Hirschhorn syndrome is caused by heterozygous chromosomal deletions that cause craniofacial malformations, intellectual disability, muscle hypotonia and heart defects [9]. This chromosomal region is also commonly deleted in bladder cancer [10]. Since a cherubism-like phenotype is not part of the Wolf-Hirschhorn syndrome, Tiziani at al. concluded that a cherubism mutation must be a gain-of-function mutation [6]. In 2001 Ueki at al. identified heterozygous mutations for cherubism in 12 families in the gene for the signaling adapter *SH3-domain binding protein 2* (*SH3BP2*) [11].

**Figure 1:**
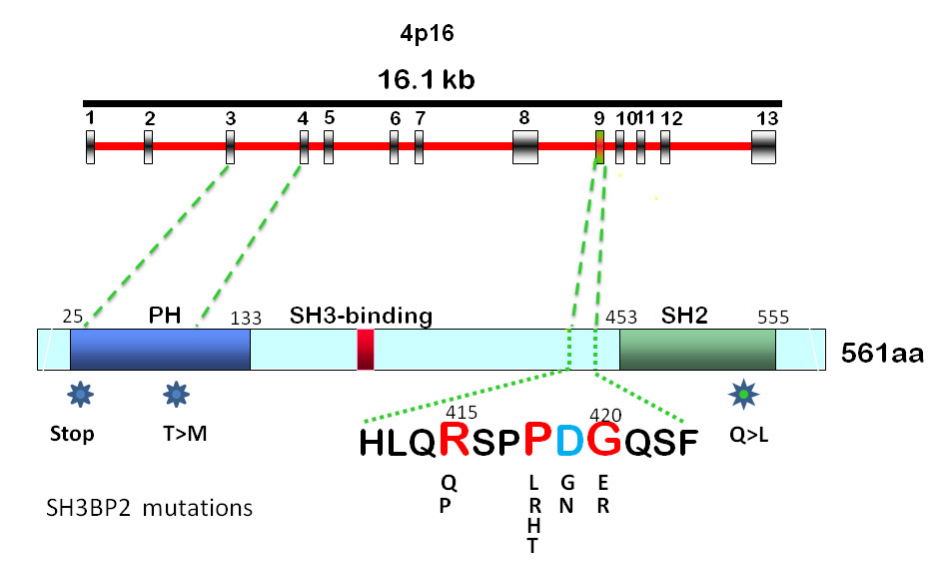
Gene map and protein structure of human SH3BP2 indicating mutations in the canonical cherubism mutation interval (amino acids 415-420) and mutations reported in the pleckstrin homology (PH) domain. The mutation in the SH2 domain has been found in tumor tissue of a patient with giant cell tumor. (Modified after Ueki et al., 2001)

SH3BP2 was initially identified as a c-Abl binding protein in mice and humans [10,12]. The *SH3BP2* gene product is expressed in most cell types. It acts as an adapter protein to control intracellular signaling by interacting and forming complexes with binding proteins [13] and with scaffolding proteins [14,15]. The 561 amino acid (aa) protein (559 aa in mouse) is highly conserved in mammals with 87% amino acid sequence homology between human and mouse [10] and 84% homology on the nucleotide level. The 48kb SH3BP2 gene contains 13 exons that code for a 62 kDa protein with 561 amino acids (Figure 1). As is the case with most adapter proteins, SH3BP2 has a modular domain structure and consists of an N-terminal pleckstrin homology (PH) domain, a proline-rich (PR) domain and a C-terminal Src-homology 2 domain (SH2). SH3BP2 is thought to bind to cell membrane lipids via its PH domain and to interact with the SH3 domains of binding partners via SH3 binding motives in the proline-rich domain. The SH2 domain can interact with a number of binding partners carrying a Tyr-Glu-Asn (YEN) binding motif (reviewed in [13]).

The mutations identified by Ueki et al. were located in exon 9, within a 6 amino acid interval (RSPPDG) in the proline-rich domain proximal to the SH2 domain of SH3BP2 (Figure 1; Table 1) [11]. All mutations were transitions or transversions of single nucleotides that led to the substitution of amino acids Arg415, Pro418 or Gly420. These mutations account for 100% of the mutations detected in the laboratory at UCHC. Additional single nucleotide substitutions were found in Gly420, Pro418 and Asp419 (Table 1; see also http://fmf.igh.cnrs.fr/ISSAID/infevers/) [16-19]. Carvalho et al. described unusual mutations in the pleckstrin homology domain in two Brazilian cherubism patients. A point mutation in exon 4 resulted in a Thr107Met substitution that was detected in blood (germline) and in tumor tissue [20]. In the tumor tissue of another patient the same group found a variant of what appears to be a deletion of nucleotide 147 (c.147delC) which led to a frame shift over 26 aa and a premature stop codon at position 325 (p.Arg49ArgfsX26) [21]. This patient suffered from a severe case of cherubism and is to our knowledge the only patient who had a fatal form of cherubism [22]. The mutation found in this patient could conceivably have led to a severe and rapidly progressing form of cherubism if the partial gene product (the N-terminal 48 amino acids) is translated. A truncated protein may have a dominant negative effect on disease mechanisms or exacerbate the disease progression by activating expression of certain (yet unknown) proteins. It is unlikely that the mutant protein is not expressed because hemizygosity, as in Wolf-Hirschhorn syndrome, is not expected to cause any cherubism-like phenotype. For all other patients with commonly detected cherubism mutations in SH3BP2 seen in our clinics or in the research laboratory we were unable to establish any genotype – phenotype correlation.

**Table 1:** Mutations in SH3BP2

Nucleotide change	Amino acid change	Exon	Phenotype	Detection	Literature
c.1244G>C	p.Arg415Pro	9	cherubism	germline	Ueki et al. (2001)
c.1244G>A	p.Arg415Gln	9	cherubism	germline	Ueki et al. (2001)
c.1253C>T	p.Pro418Leu	9	cherubism	germline	Ueki et al. (2001)
c.1253C>G	p.Pro418Arg	9	cherubism	germline	Ueki et al. (2001)
c.1253C>A	p.Pro418His	9	cherubism	germline	Ueki et al. (2001)
c.1252C>A	p.Pro418Thr	9	cherubism	germline	de Lange et al. (2007)
c.1256A>G	p.Gln419Gly	9	cherubism	germline	Li and Yu (2006)
c.1255G>A	p.Asp419Asn	9	cherubism	germline	Lietman et al. (2006)
c.1258G>C	p.Gly420Arg	9	cherubism	germline	Ueki et al. (2001)
c.1258G>A	p.Gly420Arg	9	cherubism	germline	Lo et al. (2001)
c.1259G>A	p.Gly420Glu	9	cherubism	germline	Ueki et al. (2001)
c.147delCtranslation stop at nt325 (TGA)	p.Arg49ArgfsX26	3	severe cherubism	germline	Carvalho et al. (2008)
c.320C>T	p.Thr107Met	4	cherubism	germline	Carvalho et al. (2009)
c.1442A>T	p.Gln481Leu	11	giant cell granuloma	somatic	Carvalho et al. (2009)

Cherubism-like multilocular cysts can also be found in Noonan-like/multiple giant-cell lesion syndrome [23], which is now considered part of the Noonan spectrum of phenotypes (NS/MGCLS) (NLS; MIM 163950) [24-26]. Characteristic features of Noonan syndrome include short stature, webbed neck, craniofacial malformations, cardiac abnormalities and cryptorchidism. There is considerable phenotypic variability and cherubism-like cysts that occur unilaterally or bilaterally in the mandible or maxilla or in other mineralized or soft tissues can be part of the Noonan spectrum. Mutations in NS/MGCLS have been found in the SHP2-coding gene *PTPN11* and in *SOS1 *[24,27-31]. Both gene products act in the RAS-mitogen-activated protein kinase signaling pathway and it is therefore conceivable that SH3BP2 may also play a role in this pathway. It may be worthwhile to test whether those patients who were diagnosed with cherubism and were negative for a mutation in *SH3BP2* have mutations in other genes within the RAS-MAPK axis. Interestingly, bilateral mandibular cherubism-like lesions and giant cell lesions in the mandible and in long bones have been described in neurofibromatosis patients [32,33], and are associated with mutations in the neurofibromin gene, *NF1*. NF1 is known as a regulator of the RAS pathway and mutations in *NF1* are associated with neurofibromatosis and Noonan syndrome [34,35].

To date there is only one report of a somatic mutation of *SH3BP2* in a central giant cell lesion (CGCL) [20]. The described mutation is not identical with canonical cherubism mutations in exon 9 but is a point mutation in exon 11 leading to a Glutamine 481 to Leucine exchange in the SH2 domain of SH3BP2.

Alternative splicing variants of *SH3BP2* have been identified experimentally and by computational delineations. However, it is not known whether any of these variants are biologically relevant [10,36] (see also http://genecards.org). Regulation of *SH3BP2* transcription is largely unknown but recently evidence emerged that *SH3BP2* expression is differentially regulated by hypoxic conditions in tumor cells [37]. More is known about the role its gene product plays during immune response.

## SH3BP2 function in immune cells

Before its identification as the principal disease-causing gene for cherubism, *SH3BP2* had been of interest to immunologists because of its multiple roles in hematopoietic and immune cells. Therefore a number of aliases (*SH3-domain binding protein 2; SH3BP2; 3BP2; CRBM; CRPM; RES4-23; FLJ42079; FLJ54978*) and various protein names (SH3BP2; Abl-SH3 binding protein 2; TNFAIP3 interacting protein 2) can be found in the literature.

Early investigations examined the function of SH3BP2 in hematopoietic cells and found that SH3BP2 induced B cell receptor activation, NK cell mediated cytotoxicity and basophilic cell degranulation [38-43]. The modular structure of SH3BP2 suggests that it may function as an adaptor protein [11,39,40,44] particularly as it lacks known catalytic activity. In various studies, investigators have examined the proteins that interact with SH3BP2 to derive clues about its function(s). A direct interaction between SH3BP2 and Syk was identified in a yeast 2-hybrid screen of a T lymphocyte library for Syk kinase-interacting proteins, and the role of SH3BP2 in modulating Syk activity has been examined in lymphocytes and Jurkat TAg cells [44]. In lymphocytes, SH3BP2 binds to 14-3-3, Vav1 and 2 and PLCγ1 [40,44]. In addition, an SH3BP2 mutant incapable of binding to 14-3-3 showed increased NFAT (nuclear factor of activated T cells) activation, indicating that the interaction of 14-3-3 with SH3BP2 can block its function [40]. Vav proteins are guanine nucleotide exchange factors that activate the small GTPases Ras and Rac1, which in turn activate AP-1 and NFAT, respectively [39,40,45,46]. Vav1 and Vav2 functionally cooperate with SH3BP2 in Jurkat TAg cells [39] and Vav3 is known to regulate osteoclast function [45,47].

Cbl and the Cbl interacting protein CIN85 have also been identified as proteins which directly or indirectly bind to SH3BP2 [15,44]. Cbl expression is enriched in the podosome belt in osteoclasts at sites of cell attachment and as a result c-Cbl^-/-^ osteoclasts have impaired motility [48]. CIN85 overexpression decreases intracellular calcium signaling and decreases PLCγ1 and 2 phosphorylation [49].

SH3BP2 can be modified by tyrosine and serine phosphorylation and therefore alter its activity and binding properties. SH3BP2 phosphorylation of Tyr^183^ is required for interaction with Vav1 and phosphorylation of Tyr ^446^ of SH3BP2 is required for SH3BP2 interaction with the SH2 domain of Lck [39,46]. Phosphorylation of Ser^225^ and Ser^277^ are required for 14-3-3 binding, and a SH3BP2 protein lacking these serines was shown to have increased activity in Jurkat TAg cells [40]. In T cells, SH3BP2 is phosphorylated on tyrosine^448^ in response to T cell receptor stimulation and this phosphorylation is required for T cell signaling as indicated by NFAT activiation [50]. Further, phosphorylation of SHP1 phosphatase causes recruitment and dephosphorylation of SH3BP2 and termination of T cell signaling [50]. SH3BP2 phosphorylation is also induced by CD244 ligation and tyrosine^337^ phosphorylation of CD244 regulates its interaction with SH3BP2 in NK cells [51]. Mutant SH3BP2 alters the phosphorylation of other proteins. For example, replacement of amino acids Tyr^183^ and Tyr^446^ or Arg^486^, which are phosphorylation sites, with other amino acids reduces the ability of SH3BP2 to respond to signals that activate NFAT. Moreover, heterozygous and homozygous *Sh3bp2* knockin cells that contain the P416R mutation found in cherubism patients show increased phosphorylation of ERK1/2 and Syk (at Tyr^346^) after stimulation with M-CSF and RANKL [52].

In summary, SH3BP2 can be differentially phosphorylated depending on the functions it fulfills in the various immune cell types thus attracting specific protein binding partners and regulating downstream signaling pathways. In osteoclasts, another cell type of hematopoietic origin, SH3BP2 is a major regulator of bone resorption. Mutations in SH3BP2 result in osteoclasts that lead to increased bone resorption in jaws of cherubism patients, whereas in a mouse model bone resorption is more general [11,52].

## SH3BP2 in osteoclasts

The limited distribution of bone lesions in patients with cherubism is unexpected as the disorder is associated with the heterozygous germline mutations in *SH3BP2*, which is widely expressed throughout the osteoimmune system. The precise function of the six-amino acid region where most of the known mutations occur remains unclear, but recent work suggests that the cherubism missense mutations lead to a gain-of-function rather than a loss of activity [16,52,53]. Mutations in cherubism that result in a gain-of-function for SH3BP2 is consistent with prior observations that deletions of 4p16.3 in patients with Wolf-Hirschhorn syndrome, which result in loss of one copy of *SH3BP2*, do not cause a bone resorptive phenotype [54-56].

Osteoclasts are the principal bone-resorbing cells and are important regulators of bone morphogenesis and remodeling. Osteoclasts arise from hematopoietic precursors by processes that involve growth factors, cytokines, peptides, and steroid hormones. A powerful cytokine, RANKL, binds the TNFR-related protein receptor activator of NFκB (RANK; *TNFRSF11B*), that is expressed on the surface of osteoclast progenitor cells. RANKL stimulates changes in preosteoclast gene expression that induce osteoclast differentiation and result in generation of mature, bone-resorbing osteoclasts. The formation of mature osteoclasts requires RANKL, indicating that this cytokine, in addition to colony-stimulating factor 1 (CSF-1)/macrophage colony-stimulating factor (M-CSF), is a critical differentiation factor that specifies the osteoclast maturation program, and hence induction of bone resorption. Although RANKL (in conjunction with M-CSF) has been recognized as one of the key osteoclastogenic signals expressed by osteoblasts and stromal cells, the downstream signaling pathways activated by this cytokine have not been fully characterized.

RANKL induces osteoclast formation via transcription and activation of NFATc1, the master “switch” for osteoclastogenesis [57-59]. NFATc1 is activated by calcineurin, a calcium-calmodulin dependent phosphatase, via dephosphorylation, which facilitates translocation of NFATc1 into the nucleus [57-62]. In addition to NFATc1 there are other NFAT isoforms, termed NFATc2, NFATc3, and NFATc4, but these proteins are not expressed at significant levels in pre-osteoclast cells [59].

RANKL can induce intracellular calcium oscillations to activate calcineurin in bone marrow macrophages (BMMs, BMM cells) [57] and the mouse osteoclast precursor cell line RAW 264.7 [61]. However, it is increasingly clear that other signaling pathways can also increase concentrations of cytosolic Ca^2+^, and can also activate calcineurin and NFATc1. For example, membrane proteins with immunoreceptor tyrosine-based activating motifs (ITAMs), such as FcRγ1 and DAP12 interact with their own ligands as well as activated RANK to increase cytosolic Ca^2+ ^[57,63-65]. Mechanistically, activation of these immunoreceptors in concert with RANK signaling leads to phosphorylation of the ITAM domains, which in turn recruit Syk to the membrane with subsequent activation of PLCγ. Activation of PLCγ leads to the generation of IP3, which releases Ca^2+^ from the endoplasmic reticulum and thereby stimulates calcineurin-dependent dephosphorylation of NFATc1 and consequently translocation of NFATc1 into the cell nucleus [63,65].

**Figure 2:**
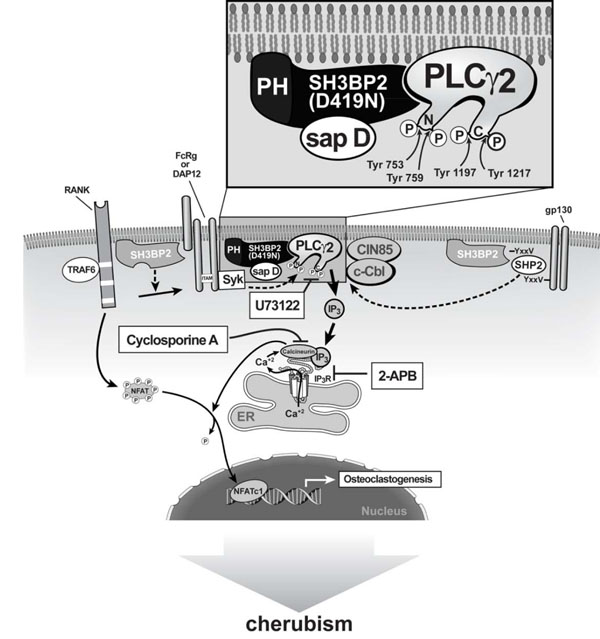
Schematic diagram of SH3BP2 interactions and pathway for SH3BP2-induced increase in osteoclastogenesis.

Overexpression of wild-type and mutant SH3BP2 in B and T cells leads to transactivation of a luciferase reporter gene that is under the control of the NFAT binding sequence from the interleukin 2 (IL-2) gene promoter [16,39,40,44]. Moreover, overexpression of a constitutively active form of NFATc1 in the RAW 264.7 osteoclast precursor cell line is sufficient to induce osteoclast differentiation [11,57,59,63]. Based on these observations Lietman and coworkers examined whether wild-type SH3BP2 increased NFAT translocation, and activation and TRAP activation in RAW 264.7 cells and whether SH3BP2 mutants found in cherubism patients further increased NFAT and TRAP activation to induce the osteoclastic bone lesions of cherubism [53,66]. Indeed, wild-type SH3BP2 increased NFAT and TRAP activation in RAW 264.7 cells [66]. This effect was dependent upon sRANKL, which induced expression of endogenous NFATc1 and was inhibited by 2-APB, U73122, and cyclosporine A, which act upstream of NFATc1 activation [57] (Figure 2). SH3BP2 specifically stimulated translocation of NFATc1 into the nucleus [66]. Moreover, isoforms of SH3BP2 carrying cherubism mutations further increased NFAT and TRAP activation and therefore these mutant forms may be a sufficient stimulus to induce the osteoclastic bone lesions of cherubism in a manner consistent with a gain-of-function mutation. At low concentrations, mutant SH3BP2 led to higher increases of NFATc1 than wild-type SH3BP2 until NFAT activity reached a plateau, which suggests that mutant SH3BP2 is more efficient in inducing osteoclastogenesis [67].

Because nuclear translocation of NFAT requires dephosphorylation by calcineurin, one may hypothesize that SH3PB2, which lacks catalytic activity, requires intermediaries to stimulate calcineurin activity. One such candidate is the SH3BP2 binding partner PLCγ. PLCγ1 is phosphorylated by sRANKL [15,39,66,68]. PLCγ, as well as other forms of PLC, cleave the membrane phospholipid phosphatidyl inositol-4,5-biphosphate (PIP2) into the second messenger molecules inositol-1,4,5-triphosphate (IP3) and diacylglycerol (DAG) [69]. IP3 directly increases intracellular calcium levels by inducing the release of endoplasmic reticulum calcium stores, which leads to activation of calcineurin. There are two forms of PLCγ (1 and 2) [68,70-72]. While PLCγ1 is widely distributed, expression of PLCγ2 is primarily limited to cells of hematopoietic lineage [70]. Both PLCγ isoforms require phosphorylation on specific tyrosine residues for their catalytic activity [71].

Targeted deletion of *Plcγ2* but not *Plcγ1* in mice results in an *in vivo* osteopetrotic phenotype [68], suggesting that PLCγ2 is the critical isoform for sRANKL-induced osteoclastogenesis. PLCγ2 has four tyrosine phosphorylation sites (Tyr^753^, Tyr^759^, Tyr^1197^, Tyr^1217^) [73-75]. In separate experiments the mutation of all four of these tyrosines had a dramatic effect on PLCγ2 activation as measured by intracellular calcium mobilization in B cells [73]. Forced expression of wild-type and mutant SH3BP2 in RAW 264.7 cells led to an increase in the relative amount of both phospho-PLCγ1 and phospho-PLCγ2, with no alteration in the total amount of either protein, and mutant SH3BP2 was more active than the wild-type [57,63,76]. Overexpression of SH3BP2 also augmented sRANKL-dependent phosphorylation of SYK, but there were no differences between wild-type and mutant SH3BP2 proteins in SYK phosphorylation. However in the SH3BP2 knockin mouse there were increases in SYK phosphorylation relative to wild-type mice [52]. Similarly, both wild-type and mutant SH3BP2 produced comparable increases in sRANKL-induced activation of VAV3 in *in vitro* experiments, which is phosphorylated by SYK. Thus, RANKL-induced phosphorylation of all four of these interacting proteins is enhanced by SH3BP2, but under the conditions that were used to replicate cherubism i.e. low dose transfections [66], mutant SH3BP2 proteins have a specific activating effect that appears to be limited to PLCγ1 and PLCγ2. The increase of PLCγ2 phosphorylation (and by inference activation) by the mutant forms of SH3BP2 compared to the wild-type is consistent with the recent finding that PLCγ2 activation can be dependent on Tec nonreceptor kinases rather than Syk [77]. Thus the effect of mutant SH3BP2 on increased osteoclastogenesis could be downstream of Syk activation (since Syk stimulation is not further increased but PLCγ is in this in vitro model) [66]. No SH3BP2 mutant was consistently more active than the others in terms of phosphorylation of PLCγ2, and stimulation of NFAT and TRAP or TRAP staining of multinucleated cells [66] (Figure 2). Based on these findings we think that SH3BP2 functions in the cytoplasm most directly by increasing phosphorylation of PLCγ2 at critical tyrosine residues. The mechanism for the PLCγ2 activation and the NFATc1 activation by SH3BP2 remains unknown.

Our knowledge of SH3BP2 in the various cell types that contribute to the cherubism phenotype is still only fragmentary. While *in vitro* studies offer valuable insights into the regulation, modification and molecular interaction of a protein, animal models are needed to investigate disease mechanisms, which in turn can be tested by *in vitro* experiments.

## Animal models

Ueki et al., created a mouse model for cherubism by using homologous recombination to introduce a proline-to-arginine substitution in SH3BP2 codon 416 that corresponds to Pro418 in humans [52]. Knockin mice were bred into a C57Bl6/J background to avoid variability due to strain differences. Heterozygous mice looked and behaved like wild type mice on gross examination. Although heterozygous mice developed osteopenia of all bones, they did not show cherubic lesions or detectable swellings of lymph nodes as the homozygous mice did. Homozygous mice were smaller at birth and failed to thrive [52,78]. They were smaller, weighed less than wild-type littermates and had an average life span of 6 months. In contrast to heterozygous littermates they developed cystic lesions with fibrous inflammatory infiltrates in the skeleton as well as in organs such as lung and liver [52].

Cherubism occurs as an autosomal dominant (AD) trait in humans whereas mice express cherubic lesions only as homozygotes. Severe phenotypes in mouse models for autosomal dominant human disorders are frequently found only in homozygote mice [79-82]. This apparent contradiction may be due to species-specific phenotypic thresholds, genetic redundancy and lifespan.

The bone-loss phenotype in homozygous mice was manifested by significant reduction of bone volume in calvaria, jaws and long bones. Exogenous bone resorption (pitting) was especially pronounced in jaw bones and at the distal end of femurs. Excessive bone resorption at the metaphyses of long bones affected cortical as well as trabecular bone and already became apparent at young age. Static histomorphometry of long bones indicated that the number of osteoblasts in homozygous mice tripled and the number of osteoclasts doubled, which suggests a possible increase in osteoblast and osteoclast activities. In vitro studies showed that mutant osteoclasts not only respond to much lower levels of the inductive cytokines RANKL and MCSF, but respond to the signals with highly increased osteoclast numbers, increased number of nuclei per osteoclast and subsequently with greater bone resorption [52]. The increased bone resorption is attributed to increased osteoclastogenesis and resorptive activity of osteoclasts and not to increased numbers of osteoclast progenitors. Osteoclast progenitor numbers are not changed between wild-type, heterozygous and homozygous mutant mice [78].

Heterozygous and homozygous mice lack sufficient numbers of mature osteoblasts [83]. The authors investigated the ratio of mature osteoblasts to immature osteoblasts *in vivo* in crosses of *Sh3bp2*^KI/KI^ mice with mice expressing GFP driven by a 3.6 kb promoter of collagen I (indicator of immature osteoblasts; pOBCol3.6GFPtpz) to crosses with a marker for mature osteoblasts (pOBCol2.3GFPemd) [84]. They found a 3-fold increase in osteoblast perimeter to bone perimeter due to overexpression of immature osteoblasts and that the mature form of osteoblasts (2.3GFP positive) is actually almost 20% lower than in wild-type mice. Similar results were seen *in vitro* in calvarial osteoblast cell culture experiments. As a result of insufficient osteoblast differentiation, mutant osteoblasts lay down undermineralized bone matrix in the mouse model [52,83]. Gene expression profiling in mutant mice showed some important differences in mutant osteoblasts, one of which was the reduced expression of osteoprotegerin, the soluble RANKL decoy receptor. The difference in the RANKL/OPG ratio may be the reason for increased osteoclastogenesis in wild-type and in knock-in osteoclasts when co-cultured with knock-in osteoblasts [83]. The studies by both groups showed that *Sh3bp2* has different functions in osteoblasts and osteoclasts. To test the relevance of the *in vivo* and *in vitro* osteoblast studies that have been performed in the mouse model it would be interesting to study osteoclasts and osteoblasts isolated from cherubism patients.

Infiltrative lesions in bone and soft-tissue organs were rich in spindle-shaped fibroblastoid cells, macrophages and TRAP-positive multinucleated osteoclast-like cells [52] and closely resembled human cherubism lesions. Because macrophages are known to produce the pro-inflammatory cytokine tumor necrosis factor-alpha (TNF-α), the authors measured TNF-α levels in serum and in isolated peritoneal macrophage populations and discovered highly increased TNF-α levels in homozygous mice while levels in heterozygous mice and wild-type mice were not measurable. In macrophage cultures, however, the heterozygous macrophages began to secrete similarly high TNF-α levels within 2 days of culture. While studying downstream effects of increased TNF-α levels, the authors found that mutant macrophages expressed higher levels of the intracellular signaling components ERK, p38, and IқBα and showed increased phosphorylation of SYK, which is a regulator of osteoclastogenesis. Additional experiments conducted in differentiating osteoclasts showed similar results and suggested that the *Sh3bp2* mutation indeed elicits a gain-of-function effect.

To study the influence of possible immune reactions on the development of inflammatory lesions, *Sh3bp2*^KI/KI^ mice were crossed with RAG1-deficient mice, which lack B- and T cells. Mice homozygous for both mutations had the same bone phenotype and inflammatory infiltrates in bones and soft-tissue organs, which suggested that immunoregulation by B- and T-cells is not involved in the cherubism phenotype. When *Sh3bp2*^KI/KI^ mice were crossed with mice lacking the cytokine M-CSF (*op/op*) the authors could show that bone loss and tissue infiltrates were virtually non-existent but TNF-α expression was still high. This strongly suggested that macrophage differentiation in this mouse model must be regulated by an M-CSF-independent pathway. When *Sh3bp2*^KI/KI^ mice were crossed with mice that lack TNF-α, the infiltrative lesions disappeared and the bone phenotype was partially rescued, although bone marrow stromal cells from double mutants still responded with increased osteoclastogenesis to M-CSF and RANKL stimulation. The double mutant *Sh3bp2*^KI/KI^ / *TNF-α^-/-^* mice resembled heterozygote *Sh3bp2*^KI/+^ mice and had a normal life span.

**Figure 3:**
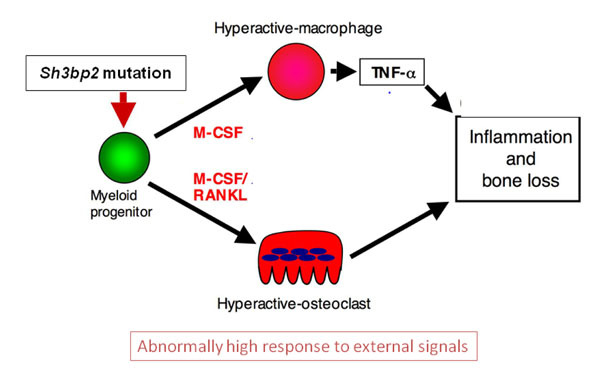
The role of TNF-α, M-CSF and RANKL in the pathogenesis of cherubism. (Modified after Ueki et al., 2007)

These results point to the existence of at least 2 mechanisms that are involved in the phenotype of the *Sh3bp2*^KI/KI^ mouse. The authors hypothesize that the effect of the mutation elicits macrophage hyper-reactivity through ERK signaling via a positive autocrine feedback loop, which leads to the increased TNF-α production and inflammatory reactions (Figure 3). The other effect is the generation of hyper-reactive osteoclasts via a Syk-related pathway that leads to increased bone resorption. While TNF-α may have a direct effect on osteoblast differentiation in vivo, there is also a cell-autonomous effect on osteoblast precursors that can be seen when mutant osteoblasts are cultured in the absence of TNF-α - producing cells [83].

As already discussed in the previous section, NFATc1 is a downstream target of RANKL signaling and a master regulator of osteoclastogenesis. The role of NFATc1 in the cherubism phenotype has been examined by crossing *Sh3bp2*^KI/KI^ mice with *Nfatc1* conditional knockout mice [85]. Cre-mediated deletion of *Nfatc1* with *Mx1-Cre* in all myeloid cells of 10-day-old mice resulted in an osteopetrotic phenotype due to lack of osteoclastogenesis. However, the skeletal *Sh3bp2*^KI/KI^ phenotype in double mutant mice was fully rescued in the absence of NFATc1 and the mice actually displayed an osteopetrosis-like phenotype. The authors showed that NFATc1 is a target of SH3bp2. NFATc1 is upregulated in RANKL/M-CSF-stimulated osteoclast precursors by mutant SH3BP2, which led to the formation of excessive numbers of osteoclasts. In the absence of NFATc1 there was no *in vitro* osteoclast formation. However, the *Sh3bp2*^KI/KI^ / *Nfatc1^-/-^* double mutants still developed inflammatory infiltrates in lungs, livers and other soft-tissue organs as TNF-α levels were still high in those mice.

These experiments confirmed that the *Sh3bp2*^KI/KI^ phenotype is caused by at least two mechanisms. Mutant SH3BP2 stimulates excessive osteoclastogenesis by increasing NFATc1 expression, which leads to increased bone resorption. Since TNF-α levels are still high in double mutants but osteoclastogenesis is disrupted, one can conclude that any effect of TNF-α on bone resorption in the cherubism model must go through NFATc1 while signs of inflammatory reactions without osteoclast involvement are independent of NFATc1. TNF-α is regulated by SH3BP2 through a mechanism not involving NFATc1 but possibly other NFAT family members [86].

Aliprantis and coworkers also showed that NFATc1 has an inhibitory function on the expression of osteoprotegerin in stimulated bone marrow osteoclast precursor cells. It is still to be determined whether the reduced level of OPG in osteoblasts of *Sh3bp2*^KI/KI^ mice [83] also depends on NFATc1.

Mice in which *Sh3bp2* was ablated showed deficiencies mainly in the adaptive immune system. *Sh3bp2* is required for functional B-cell receptor (BCR) signaling while it is not needed for T-cell receptor (TCR) signaling [38]. The delayed B-cell response may be explained in part by reduced proliferation and increased apoptosis induced by B-cell receptor signaling [87]. Investigating skeletal responses to *Sh3bp2* ablation may further illuminate the functions of *Sh3bp2* although results have not yet been made public.

While initial investigations of the cherubism mouse model focused on the skeletal phenotype and abnormal osteoclast and osteoclast differentiation, it became soon apparent that the phenotype in the *Sh3bp2*^KI/KI^ mice is at least in part based on abnormal immune response. Then, Ueki and coworkers showed that the generalized chronic inflammation in the *Sh3bp2*^KI/KI^ mouse is elicited by TNF-α and is independent of B- or T-cell involvement. The disease phenotype can be transferred by myeloid cells (monocytes, macrophages) and it can therefore be argued that the disease phenotype is mediated by abnormal innate immune response and should be included in the list of autoinflammatory diseases with known genetic origin [88].

## Cherubism as an inflammatory disorder

Autoinflammatory disorders are defined by multisystem inflammation without the production of high-titer autoantibodies or identifiable pathogens [89-91]. Cherubism fulfills these criteria in the mouse model where infiltrating inflammatory lesions are found in many organs and in human patients where bone lesions are limited to the jaws but swelling of lymph nodes is found during or prior to cherubic episodes. Because the process is (at least in the mouse) driven by high levels of TNF-α it could be argued that cherubism is as much a systemic disorder of myeloid cells as it is a matrix disorder [92]. Pro-TNF-α is a plasma membrane protein and the soluble form of TNF-α is released by matrix metalloproteinases. The various responses to membrane-associated and soluble TNF-α are elicited upon binding of TNF-α to its transmembrane receptors TNFR1 and TNFR2 and the subsequent activation of distinct signaling pathways [93].

TNF-α is also a key player in the host defense to bacterial, viral and parasitic infections [93] where it mediates the normal response to the infective agent. However, excessive TNF-α expression or a temporally or spatially inappropriate expression can have damaging effects to the organism, which results in osteopenia and infiltrative inflammatory lesions in the *Sh3bp2*^KI/KI^ mouse.

It has long been hypothesized that the limitation of bone-resorptive lesions to the jaws in human cherubism patients is connected to rapid bone remodeling during the development and eruption of the secondary dentition in children [2,11]. The bone remodeling needed in the process of tooth eruption elicits the expression and recruitment of a host of cytokines. It could be those cytokines and the hypersensitivity of myeloid cells that trigger a self-sustaining loop of TNF-α expression that leads to osteoclastogenesis, soft fibrous tissue proliferation and swollen lymph nodes. In an ongoing study, Ueki and co-workers offer a new hypothesis for the restriction of cherubism lesions to the jaws. They suspect that the trigger for cherubism in patients that are heterozygous for a *Sh3bp2* mutation could be a hyper-reactive host response to oral pathogens or physical damage that occurs on a regular basis in the oral cavity [94].

Lipopolysaccharide (LPS) produced by Gram-negative commensal bacteria is known to induce osteoclastogenesis, TNF-α expression and bone loss [95]. It is conceivable that cherubism patients are predisposed to osteolytic reactions in the jaws once a certain threshold for inducing agents (from intense bone remodeling in addition to commensal bacterial load) has been reached. LPS can enhance osteoclastogenesis in RANKL -induced osteoclast precursors [96]. LPS can also inhibit osteoblast differentiation [97,98] through the Toll-like receptor expressed on osteoblasts and its interaction with myeloid differentiation factor 88 (MyD88) [99]. The myeloid differentiation marker MyD88 is an adaptor protein that mediates host response to damage- and pathogen-associated molecular events. MyD88 is known to act downstream of Toll-like receptors and the interleukin-1 receptor by interacting with their intracellular Toll/IL-1 receptor homology domains [100]. Current literature suggests that the role of MyD88 in LPS-stimulated osteoclastogenesis is mainly via RANKL stimulation in osteoblasts and by supporting the survival of differentiated osteoclasts [101].

Ueki and coworkers are now investigating why crosses of *Sh3bp2*^KI/KI^ and MyD88 deficient mice show less inflammatory infiltrates in bone and other organs and significant improvement of facial swellings and bone resorption [94]. While the importance of LPS or other bacterial products in this partial “rescue” is not yet known, it is obvious that MyD88 plays a major role in the cherubism phenotype of the mouse model and MyD88-independent pathways are likely to contribute as well. Future research will show whether this TLR/IF-1 pathway is needed only for the early stage of cherubism to generate sufficient pro-inflammatory signals and whether some auto-stimulatory loop takes over or whether it is required to maintain the phenotype. Whatever the outcome of this exciting work in progress may be, it is likely to lead to new targets for treatment or prevention of cherubism.

This review covers the current knowledge on genetic and molecular aspects of SH3BP2 and the lessons from mouse models. While it is evident that SH3BP2 is an important player in bone remodeling in the mouse and that SH3BP2 acts through NFATc1 to stimulate osteoclastogenesis, other details of the SH3BP2/ NFATc1 axis are still elusive. Inflammatory responses elicited by the Pro416 mutation in the *Sh3bp2* knock-in mouse are independent of NFATc1 and are likely to be the major drivers for continued bone resorption. There is no current evidence that suggests that immune response in cherubism patients is abnormal. However, cherubic bone resorption is preceded or accompanied by submandibular lymph node swelling, which has not yet been thoroughly investigated. Further immunologic research is needed to study the initiation of bone resorption in the mouse model and how the extra-skeletal inflammatory infiltrations develop. The ultimate goal is to test those findings in cherubism patients and to identify ways to treat or better still, to prevent the disease.

## Abbreviations

kDa: kiloDalton; aa: amino acid; SH3BP2: src homology 3 binding protein 2; PH: pleckstrin homology domain; PR: proline-rich domain; SH2: Src-homology 2 domain; Tyr: Tyrosine; Glu: Glutamic Acid; Asn: Asparagine; NS/MGCLS: Noonan syndrome/multiple giant-cell lesion syndrome; PTPN11: gene encoding the protein tyrosine phosphatase (PTP) Shp2; SOS1: gene encoding the son of sevenless homolog 1 protein; CGCL: central giant cell lesion; NFAT: nuclear factor of activated T cells; PLCγ: phospholipase Cγ; TRAP: tartrate resistant acid phosphatase; sRANKL: soluble receptor activator of NFκB ligand; OPG: osteoprotegerin; TNF-α: tumor necrosis factor-alpha; ERK: extracellular-signal-regulated kinases; SFK: src family kinase; GFP: green fluorescent protein; Jurkat T Ag: Jurkat T Antigen; NFAT-luc: NFAT luciferase; WT: wild-type; OMIM: online mendelian inheritance in man; M-CSF: macrophage-colony stimulating factor; PKC: protein kinase C; TNFR: tumor necrosis factor receptor; BMM: bone marrow macrophages; ITAM: immunoreceptor tyrosine-based activating motifs; MYD88: myeloid differentiation primary response gene (88).

## Competing interests

The authors declare that they have no competing interests.

## Authors’ contributions

EJR and SL have drafted the manuscript. All authors were involved in the critical review of the manuscript. All authors read and approved the final manuscript.
